# Coding and Decoding with Adapting Neurons: A Population Approach to the Peri-Stimulus Time Histogram

**DOI:** 10.1371/journal.pcbi.1002711

**Published:** 2012-10-04

**Authors:** Richard Naud, Wulfram Gerstner

**Affiliations:** School of Computer and Communication Sciences and School of Life Sciences, Brain Mind Institute, Ecole Polytechnique Fédérale de Lausanne, Lausanne-EPFL, Lausanne, Switzerland; Indiana University, United States of America

## Abstract

The response of a neuron to a time-dependent stimulus, as measured in a Peri-Stimulus-Time-Histogram (PSTH), exhibits an intricate temporal structure that reflects potential temporal coding principles. Here we analyze the encoding and decoding of PSTHs for spiking neurons with arbitrary refractoriness and adaptation. As a modeling framework, we use the spike response model, also known as the generalized linear neuron model. Because of refractoriness, the effect of the most recent spike on the spiking probability a few milliseconds later is very strong. The influence of the last spike needs therefore to be described with high precision, while the rest of the neuronal spiking history merely introduces an average self-inhibition or adaptation that depends on the expected number of past spikes but not on the exact spike timings. Based on these insights, we derive a ‘quasi-renewal equation’ which is shown to yield an excellent description of the firing rate of adapting neurons. We explore the domain of validity of the quasi-renewal equation and compare it with other rate equations for populations of spiking neurons. The problem of decoding the stimulus from the population response (or PSTH) is addressed analogously. We find that for small levels of activity and weak adaptation, a simple accumulator of the past activity is sufficient to decode the original input, but when refractory effects become large decoding becomes a non-linear function of the past activity. The results presented here can be applied to the mean-field analysis of coupled neuron networks, but also to arbitrary point processes with negative self-interaction.

## Author Summary

How can information be encoded and decoded in populations of adapting neurons? A quantitative answer to this question requires a mathematical expression relating neuronal activity to the external stimulus, and, conversely, stimulus to neuronal activity. Although widely used equations and models exist for the special problem of relating external stimulus to the action potentials of a single neuron, the analogous problem of relating the external stimulus to the activity of a *population* has proven more difficult. There is a bothersome gap between the dynamics of single adapting neurons and the dynamics of populations. Moreover, if we ignore the single neurons and describe directly the population dynamics, we are faced with the ambiguity of the adapting neural code. The neural code of adapting populations is ambiguous because it is possible to observe a range of population activities in response to a given instantaneous input. Somehow the ambiguity is resolved by the knowledge of the population history, but how precisely? In this article we use approximation methods to provide mathematical expressions that describe the encoding and decoding of external stimuli in adapting populations. The theory presented here helps to bridge the gap between the dynamics of single neurons and that of populations.

## Introduction

**Figure 1:**
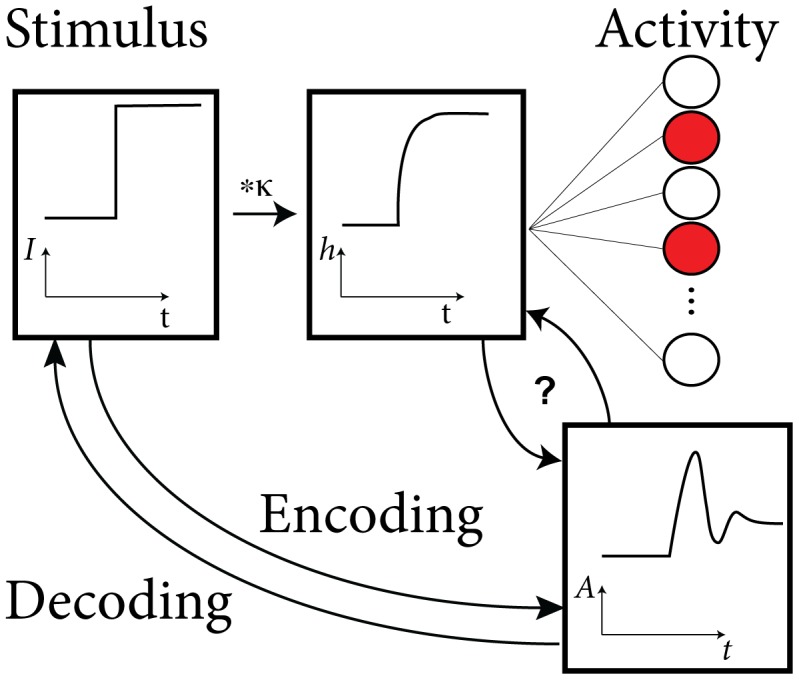
Encoding and Decoding with neuronal populations. What is the function that relates an arbitrary stimulus to the population activity of adapting neurons? We focus on the problem of relating the filtered input 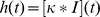 to the activity 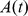. The population activity is the fraction of active neurons (red) in the population of neurons (right). All neurons are identical and receive the same stimulus. One possible stimulus 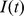 is a step current (left).

Encoding and decoding of information with populations of neurons is a fundamental question of computational neuroscience [1]–[3]. A time-varying stimulus can be encoded in the active fraction of a population of neurons, a coding procedure that we will refer to as population coding (Fig. 1). Given the need for fast processing of information by the brain [4], population coding is an efficient way to encode information by averaging across a pool of noisy neurons [5], [6] and is likely to be used at least to some degree by the nervous system [7]. For a population of identical neurons, the instantaneous population firing rate is proportional to the Peri-Stimulus Time Histogram (PSTH) of a single neuron driven repeatedly by the same stimulus over many trials.

When driven by a step change in the input, the population of neurons coding for this stimulus responds first strongly but then adapts to the stimulus. To cite a few examples, the activity of auditory nerve fibers adapt to pure tones [8], cells in the retina and the visual cortex adapt to contrast [9], [10] and neurons in the inferior temporal cortex adapt to higher order structures of images [11]. Adaptation is energy-efficient [12] but leads to a potentially ambiguous code because adapting responses generate a population activity which does not directly reflect the momentary strength of the stimuli [13]. Putting the case of sensory illusions aside, the fact that our perception of constant stimuli does not fade away indicates that the adapting responses can be efficiently decoded by the brain areas further down the processing stream. In fact, illusions such as the motion after-effect are believed to reflect errors in decoding the activity of neuronal populations [14]. But what *is* the correct rule to decode population activity? What elements of the population history are relevant? What are the basic principles?

Synapse- and network-specific mechanisms merge with intrinsic neuronal properties to produce an adapting population response. Here we focus on the intrinsic mechanisms, commonly called spike-frequency adaptation. Spike-frequency adaptation appears in practically all neuron types of the nervous system [15]. Biophysical processes that can mediate spike-frequency adaptation include spike-triggered activation/inactivation of ion-channels [16]–[18] and a spike-triggered increase in the firing threshold [19]–[22]. Neurons adapt a little more each time they emit a spike, and it is the cumulative effect of all previous spikes that sets the level of adaptation. The effect of a single spike on future spiking probability cannot be summarized by a single time constant. Rather, the spike-triggered adaptation unfolds on multiple time scales and varies strongly across cell-types [22], [23].

Mean-field methods were used to describe: attractors [24]–[28], rapid-responses [6], [29] and signal propagation [30]. While adaptation is important to correctly predict the activity of single neurons [22], [31]–[33], it is difficult to include it in mean-field methods. A theory relating spike-frequency adaptation to population dynamics should be general enough to encompass a variety of different spike-triggered adaptation profiles, as observed in experiments. In the literature we find six main approaches to the population coding problem. The first and most simple one formulates the rate of a neuronal population (or the time-dependent rate in a PSTH) as a linear function of the stimulus. This phenomenological model relates to the concept of receptive fields [34] and can be made quantitative using a Wiener expansion [35]. Yet, early experimental tests showed that linear filtering must be complemented with a non-linear function [35], [36]. The linear-non-linear model can thus be considered as the second approach to population coding. In combination with a Poisson spike generator it is called the LNP model for Linear-Nonlinear-Poisson. It makes accurate predictions of experimental measurements for stationary stimulus ensembles, but fails when the stimulus switches either its first or second order statistics. Neural refractoriness is in part responsible for effects not taken into account in this linear-nonlinear model [37]–[40]. In a third approach proposed by Wilson and Cowan [41] the population activity is the solution to a non-linear differential equation. Unfortunately this equation has only a heuristic link to the underlying neuronal dynamics and cannot account for rapid transients in the population response. The fourth approach formulates the population activity in terms of an integral equation [6], [41], [42] which can be interpreted as a (time-dependent) renewal theory. While this renewal theory takes into account refractoriness (i.e. the effect of the most recent spike) and captures the rapid transients of the population response and PSTH, neither this one nor any of the other encoding frameworks mentioned above consider adaptive effects. To include adaptation into previously non-adaptive models, a common approach is to modify the effective input by rescaling the external input with a function that depends on the mean neuronal firing rate in the past [15], [43], [44]. This forms the fifth method. For example, Benda and Herz [15] suggested a phenomenological framework in which the linear-non-linear approach is modified as a function of the past activity while Rauch et al. [43] calculated the effective rate in integrate-and-fire neurons endowed with a frequency-dependent modification of the input current. Finally, there is also a sixth method to determine the population activity of adapting populations. Inspired by the Fokker-Planck approach for integrate-and-fire neurons [27], this last approach finds the population activity by evolving probability distributions of one or several state variables [45]–[49]. Isolating the population activity then involves solving a non-linear system of partial differential equations.

The results described in the present article are based on two principal insights. The first one is that adaptation reduces the effect of the stimulus primarily as a function of the expected number of spikes in the recent history and only secondarily as a function of the higher moments of the spiking history such as spike-spike correlations. We derive such an expansion of the history moments from the single neuron parameters. The second insight is that the effects of the refractory period are well captured by renewal theory and can be superimposed on the effects of adaptation.

The article is organized as follows: after a description of the population dynamics, we derive a mathematical expression that predicts the momentary value of the population activity from current and past values of the input. Then, we verify that the resulting encoding framework accurately describes the response to input steps. We also study the accuracy of the encoding framework in response to fluctuating stimuli and analyze the problem of decoding. Finally, we compare with simpler theories such as renewal theory and a truncated expansion of the past history moments.

## Results

To keep the discussion transparent, we focus on a population of unconnected neurons. Our results can be generalized to coupled populations using standard theoretical methods [3], [6], [27].

### Encoding Time-dependent Stimuli in the Population Activity

How does a population of adapting neurons encode a given stimulating current 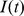
? Each neuron in the population will produce a spike train, denoted by 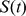, such that the population can be said to respond with a set of spike trains. Using the population approach, we want to know how the stimulus is reflected in the fraction of neurons that are active at time 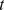, that is, the population activity 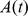 (Fig. 1). The population activity (or instantaneous rate of the population) is a biologically relevant quantity in the sense that a post-synaptic neuron further down the processing stream receives inputs from a whole population of presynaptic neurons and is therefore at each moment in time driven by the spike arrivals summed over the presynaptic population, *i. e.* the presynaptic population activity.

Mathematically, we consider a set of spike trains in which spikes are represented by Dirac-pulses centered on the spike time 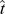: 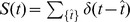 [3]. The population activity is defined as the expected proportion of active neurons within an infinitesimal time interval. It corresponds, in the limit of a large population and small time interval, to the number of active neurons 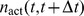 in the time interval 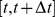 divided by the total number of neurons 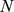 and the time interval 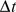 [3]:
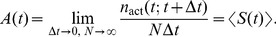
(1)The angular brackets 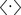 denote the expected value over an ensemble of identical neurons. Experimentally, the population activity is estimated on a finite time interval and for a finite population. Equivalently the population activity can be considered as an average over independent presentations of a stimulus in only one neuron. In this sense, the population activity is equivalent to both the time-dependent firing intensity and the Peri-Stimulus Time Histogram (PSTH).

Since the population activity represents the instantaneous firing probability, it is different from the conditional firing intensity, 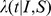, which further depends on the precise spiking history, or past spike train 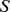. Suppose we have observed a single neuron for a long time (e.g. 10 seconds). During that time we have recorded its time dependent input current 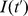 and observed its firing times 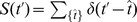. Knowing the firing history 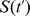 for 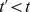 and the time-dependent driving current 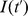 for 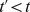, the variable 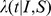 describes the instantaneous rate of the neuron to fire again at time 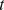. Intuitively, 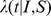 reflects a likelihood to spike at time 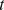 for a neuron having a specific history while 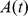 is the firing rate at time 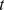 averaged on all possible histories (see Methods):
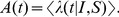
(2)


Ideally, one could hope to estimate 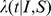 directly from the data. However, given the dimensionality of 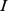 and 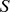, model-free estimation is not feasible. Instead we use the Spike Response Model (SRM; [6], [50]–[52]), which is an example of a Generalized Linear Model [53], in order to parametrize 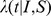, but other parametrizations outside the exponential family are also possible. In particular, 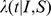 can also be defined for nonlinear neuron models with diffusive noise in the input, even though explicit expressions are not available. The validity of the SRM as a model of neuronal spike generation has been verified for various neuron types and various experimental protocols [22], [31], [32]. In the SRM, the conditional firing intensity 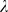 increases with the effective input 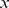:
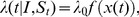
(3)where 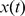 is the total driving force of the neuron:
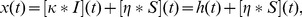
(4)where ‘
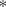
’ denotes the convolution, 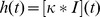 is the input current convolved with 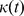 the membrane filter, 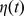 encodes the effect of each spike on the probability of spiking, 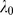 is a scaling constant related to the instantaneous rate at the threshold with units of inverse time (see Methods for model parameters). The link-function 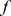 can take different shapes depending on the noise process [3]. Here we will use an exponential link-function since it was shown to fit the noisy adaptive-exponential-integrate-and-fire model [54] as well as experimental data [22], [32], [55]. The exponential link-function: 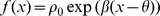 corresponds to 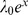 after absorbing the scaling parameter 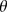 in the constant 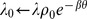 and 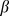 and in the functions 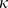 and 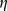 to make these unit-free.

To see that the function 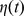 can implement both adaptation and refractoriness, let us first distinguish these processes conceptually. The characteristic signature of refractoriness is that the interspike interval distribution for constant input is zero or close to zero for very short intervals (e.g. one millisecond) - and in the following we use this characteristic signature as a definition of refractoriness. With this definition, a Hodgkin-Huxley model (with or without noise) or a leaky integrate-and-fire model (with or without diffusive noise) are refractory, whereas a Linear-Nonlinear-Poisson Model is not. In fact, every neuron model with intrinsic dynamics exhibits refractoriness, but Poissonian models do not.

While refractoriness refers to the interspike-interval distribution and therefore to the dependence upon the *most recent* spike, adaptation refers to the effect of multiple spikes. Adaptation is most clearly observed as a successive increase of interspike intervals in response to a step current. In contrast, a renewal model [56], where interspike intervals are independent of each other, does not exhibit adaptation (but does exhibit refractoriness). Similarly, a leaky or exponential integrate-and-fire model with diffusive noise does not show adaptation. A Hodgkin-Huxley model with the original set of parameters exhibits very little adaptation, but addition of a slow ion current induces adaptation.

Conceptually, contributions of multiple spikes must accumulate to generate spike frequency adaptation. In the Spike Response Model, this accumulation is written as a convolution: 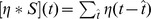. If 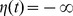 for 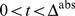 and vanishes elsewhere, the model exhibits absolute refractoriness of duration 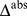 but no adaptation. If 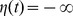 for 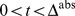 and 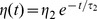 with 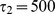 ms, then the model exhibits adaptation in addition to refractoriness. In all the simulations, we use 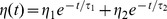 with 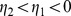 and 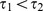, With this choice of 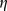 we are in agreement with experimental results on cortical neurons [22], but the effects of adaptation and refractoriness cannot be separated as clearly as in the case of a model with absolute refractoriness. Loosely speaking, the long time constant 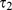 causes adaptation, whereas the short time constant 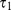 mainly contributes to refractoriness. In fact, for 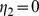 and 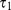 equal to the membrane time constant, the model becomes equivalent to a leaky integrate-and-fire neuron [3], so that the neuron is refractory and non-adapting. In the simulations, 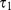 is longer than the membrane time constant so that, for very strong stimuli, it may also contribute to adaptation. We note that the formalism developed in this paper does not rely on our specific choice of 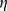. We only require (i) causality by imposing 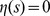 for 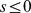 and (ii) 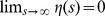 so that the effect of a past spike decreases over time.

The effects described by 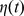 can be mediated by a dynamic threshold as well as spike-triggered currents [22]. Throughout the remainder of the text we will refer to 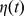 as the effective spike after-potential (SAP). It is, however, important to note that 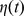 has no units, *i.e.* it relates to an appropriately scaled version of the experimentally measured spike after-potential. A depolarizing (facilitating) SAP is associated with 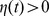, while a hyperpolarizing (adapting) SAP is associated with 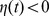.

### Quasi-Renewal Theory

In a population of neurons, every neuron has a different spiking history defined by its past spike train 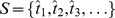 where 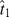 is the most recent spike, 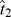 the previous one and so on. To find the population activity at any given time, we hypothesize that the strong effect of the most recent spike needs to be considered explicitly while the rest of the spiking history merely introduces a self-inhibition that is similar for all neurons and that depends only on the average firing profile in the past. Thus for each neuron we write the past spike train as 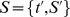 where 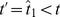 is the time of the *last* spike. Our hypothesis corresponds to the approximation 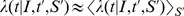, *i.e.* the last spike needs to be treated explicitly, but we may average across earlier spike times. This approximation is not appropriate for intrinsically bursting neurons, but it should apply well to other cell types (fast-spiking, non-fast-spiking, delayed, low-threshold). According to this hypothesis, and in analogy to the time-dependent renewal theory [3], [42] we find (derivation in Methods):
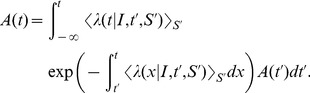
(5)Unfortunately Eq. 5 remains insolvable, because we do not know 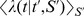. Using Eqs. 3 and 4 we find:
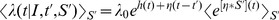
(6)As mentioned above, we hypothesize that the spiking history before the previous spike merely inhibits subsequent firing as a function of the average spiking profile in the past. In order to formally implement such an approximation, we introduce a series expansion [57] in terms of the spiking history moments (derivation in Methods) where we exploit the fact that 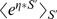 is a moment generating function:
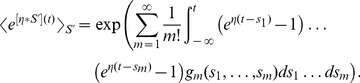
(7)The first history moment 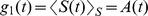 relates to the expected number of spikes at a given time 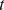. The second history moment considers the spike-spike correlations 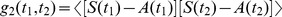 and so on for the higher moments.

We truncate the series expansion resulting from Eq. 7 at the first order (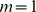). We can then write Eq. 6 as:
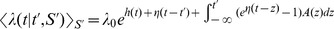
(8)We can insert Eq. 8 in Eq. 5 so as to solve for 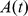 as a function of the filtered input 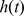. The solutions can be found using numerical methods.

We note that by removing the integral of 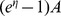 from Eq. 8 we return exactly to the renewal equation for population activity (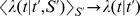). Adaptation reduces the driving force by an amount proportional to the average spike density before 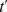, that is, the average spiking density before the most recent spike. In other words, instead of using the specific spike history of a given neuron, we work with the average history except for the most recent spike which we treat explicitly. We call Eqs. 5 and 8 the Quasi-Renewal equation (QR) to acknowledge its theoretical foundations. It is renewal-like, yet, we do not assume the renewal condition since a new spike does not erase the effect of the previous history (see Methods).

### Encoding and Decoding Time-Dependent Stimuli

Let us now assess the domain of validity of the QR theory by comparing it with direct simulations of a population of SRM neurons. To describe the single neurons dynamics, we use a set of parameters characteristic of L2–3 pyramidal cells [22]. The SAP is made of two exponentials: one with a short time constant (30 ms) but large amplitude and another with a long time constant (400 ms) but a small amplitude. The results presented here are representative of results that can be obtained for any other physiological set of parameters. For details on the simulation, see Methods.

**Figure 2:**
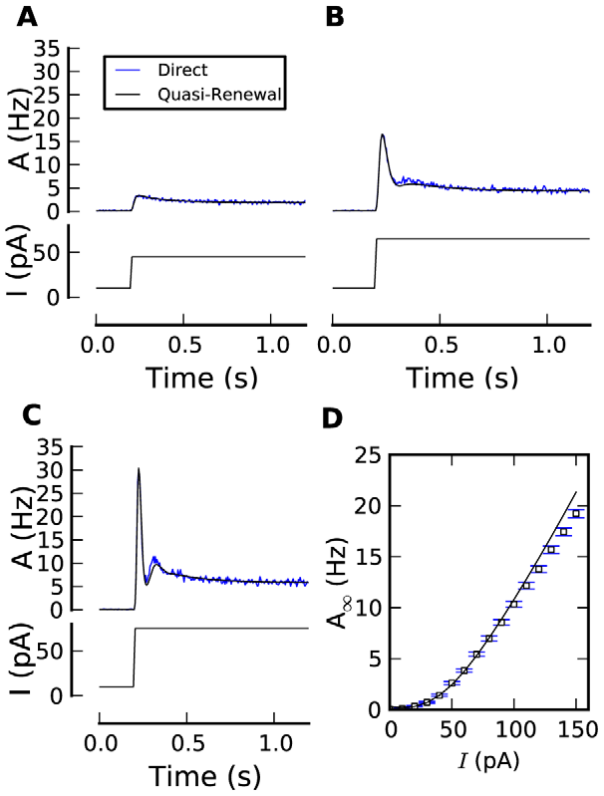
Quasi-renewal theory for step responses with realistic SAP. (**A–C**) Population activity responses (top panels; PSTH from 25,000 repeated simulations in blue, quasi-renewal theory in black) to the step current input as shown in bottom panels (black). The input step size was increased from **A** to **C**. The mean first and last interspike interval were 458
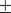
2 ms and 504
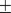
2 ms, respectively, in **A**, 142.1
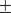
0.4 and 214
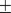
1 ms in **B**, 93.5
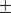
0.2 and 163.2
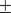
0.5 ms in **C**. (**D**) Steady-state activity vs. input current for simulations of 25,000 independent neurons (blue) and quasi-renewal theory (black). The SAP was fixed to 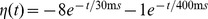 [22]. For other model parameters see Models and Methods.

The response to a step increase in stimulating current is a standard paradigm to assess adaptation in neurons and used here as a qualitative test of our theory. We use three different step amplitudes: weak, medium and strong. The response of a population of, say, 25,000 model neurons to a *strong* step increase in current starts with a very rapid peak of activity. Indeed, almost immediately after the strong stimulus onset, most of the neurons are triggered to emit a spike. Immediately after firing at 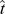, the membrane potential of theses neurons is reset to a lower value by the contribution of the SAP; 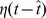. The lower membrane potential leads to a strong reduction of the population activity. Neurons which have fired at time 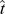 are ready to fire again only after the SAP has decreased sufficiently so that the membrane potential can approach again the threshold 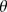. We can therefore expect that a noiseless population of neurons will keep on oscillating with the intrinsic firing frequency of the neurons [6]; however, due to stochastic spike emission of a noisy population the neurons in the population gradually de-synchronize. The damped-oscillation that we see in response to a strong step stimulus (Fig. 2C) is the result of this gradual de-synchronization. Similar damped oscillations at the intrinsic firing frequency of the neurons have also been observed for a Spike Response Model with renewal properties [6], i.e., a model that only remembers the effect of the last spike.

In contrast to renewal models (i.e., models with refractoriness but no adaptation), we observe in Fig. 2C that the population activity decays on a slow time scale, taking around one second to reach a steady state. This long decay is due to adaptation in the single-neuron dynamics, here controlled by the slow time constant 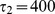 ms. The amount of adaptation can be quantified if we compare, for a given neuron its first interspike interval after stimulus onset with the last interspike interval. The mean first interspike interval (averaged over all neurons) for the strong step stimulus is 93 ms while the last interval is nearly twice as long (163 ms), indicating strong adaptation. For smaller steps, the effect of refractoriness is less important so that adaptation becomes the most prominent feature of the step response (Fig. 2A). An appropriate encoding framework should reproduce both the refractoriness-based oscillations and the adaptation-based decay.

The QR equation describes well both the damped oscillation and the adapting tail of the population activity response to steps (Fig. 2). Also, the steady state activity is predicted over a large range (Fig. 2D). We note that an adaptation mechanism that is essentially subtractive on the membrane potential (Eq. 4) leads here to a divisive effect on the frequency-current curve. Altogether, we conclude the QR theory accurately encode the response to step stimulus.

**Figure 3:**
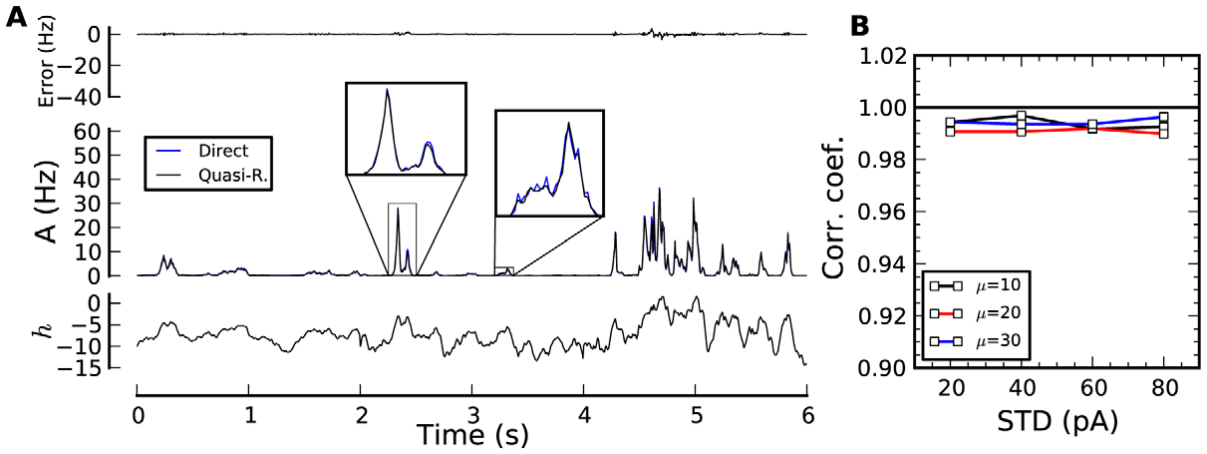
Encoding time-dependent stimuli in the population activity. (**A**) Population activity responses (middle panel; PSTH from 25,000 repeated simulations in blue, quasi-renewal theory in black) to the time-dependent stimuli shown in the bottom panel (black). The difference between direct simulation and theory is shown in the top panel. The stimulus is an Ornstein-Uhlenbeck process with correlation time constant of 300 ms, a STD increasing every 2 seconds (20,40,60 pA) and a mean of 10 pA. (**B**) Correlation coefficients between direct simulation and QR for various STDs and mean (in pA) of the input current.

Step changes in otherwise constant input are useful for qualitative assessment of the theory but quite far from natural stimuli. Keeping the same SAP as in Fig. 2, we replace the piecewise-constant input by a fluctuating current (here Ornstein-Uhlenbeck process) and study the validity of QR over a range of input mean and standard deviation (STD), see Fig. 3. As the STD of the input increases, the response of the population reaches higher activities (maximum activity at STD = 80 pA was 89 Hz). The prediction by the QR theory is almost perfect with correlation coefficients consistently higher than 0.98. Note that the correlation coefficient is bounded above by the finite-size effects in estimating the average of the 25,000-neuron simulation. Over the range of input studied, there was no tendency of either overestimating or underestimating the population activity (probability of positive error was 0.5). There was only a weak tendency of increased discrepancies between theory and simulation at higher activity (correlation coefficient between simulated activity and mean square error was 0.25).

**Figure 4:**
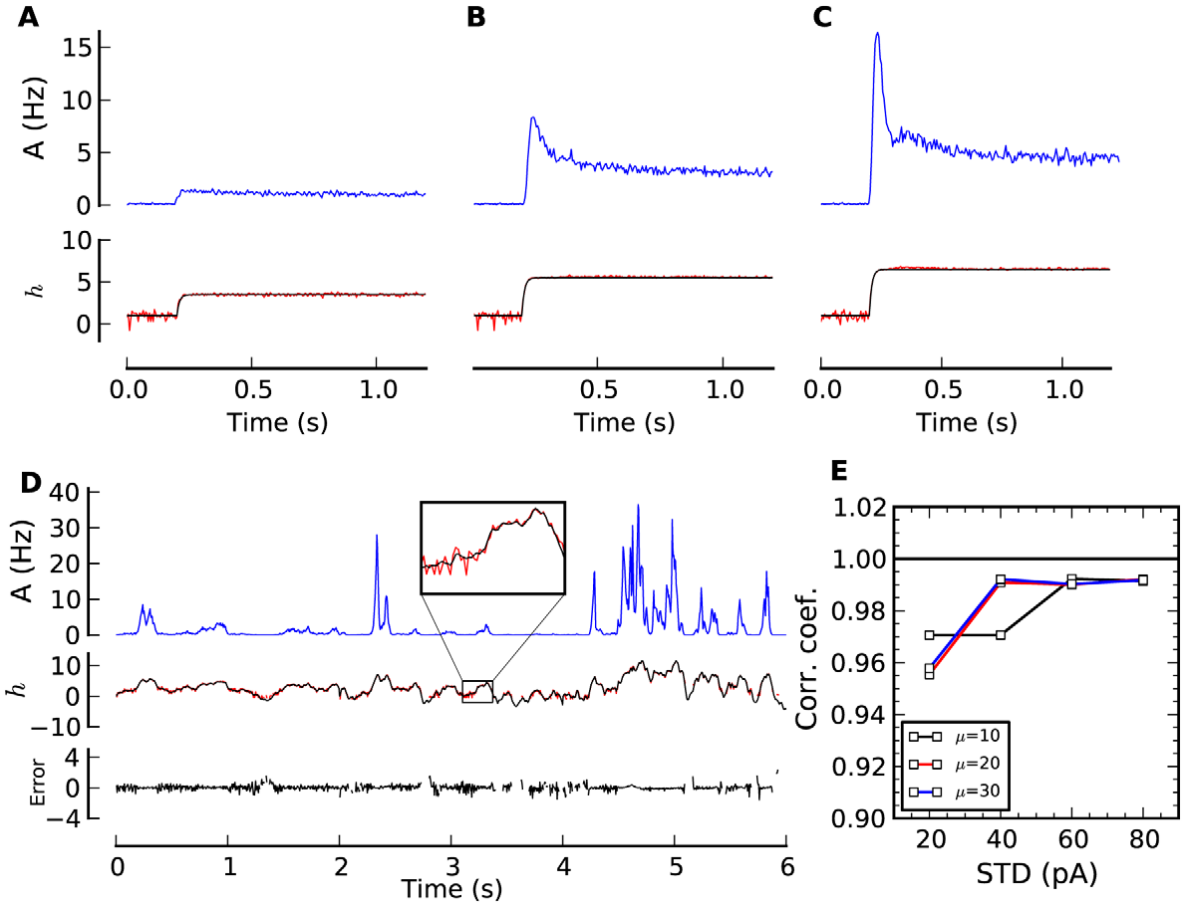
Decoding the stimulus from the population activity. (**A**–**C**) The original (bottom panels, black line) and decoded stimulus (bottom panels, red line; arbitrary units) recovered from the PSTH of 25,000 independent SRM neurons (top panels; blue line) with the QR decoder (Eq. 45). (**D**) Same as before but for time-dependent input. The decoded waveform of negative input is occasionally undefined and corresponds to input outside the dynamic range. The difference between direct simulation and theory is shown in the bottom panel. (**E**) Correlation coefficient between original and decoded input as a function of input STD, shown for three distinct mean input (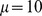 pA, 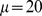 pA, and 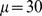 pA). Decoding based on quasi-renewal theory (Methods).

Decoding the population activity requires solving the QR equation (Eq. 5 and 8) for the original input 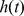 (see Methods). Input steps can be correctly decoded (Fig. 4A–C) but also fluctuating stimuli (Fig. 4D–E). Again, the input mean does not influence the precision of the decoding (Fig. 4E). The numerical method does not decode regions associated with population activities that are either zero or very small. Accordingly, the correlation coefficient in Fig. 4E is calculated only at times where decoding could be carried out. Note that unless one is to estimate the statistics of the input current and assume stationarity, it is impossible for any decoder to decode at times when 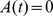. If the size of the population is decreased, the performance of the QR decoder decreases (Fig. S1). Finite size effects limit decoding performance by increasing the error on the mean activity (as can be seen by comparing the effect of filtering the average population activity (Fig. S1A and B)). Another finite-size effect is that at small population sizes there is a greater fraction of time where an estimate of the activity is zero and the decoding cannot be performed (Fig. S1D–F). Also, decoding errors are larger when 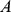 is close to zero (Fig. S1C). Nevertheless, for an input with STD = 40 pA and a population of 250 neurons, QR decoding can be performed 55% of the times with a correlation coefficient of 0.92. If the filtering of the population activity is on a longer time scale (20 ms instead of 2 ms) then decoding is possible 82% of the times and the accuracy is roughly the same (Fig. S1).

### Comparing Population Encoding Theories

We will consider two recent theories of population activity from the literature. Both can be seen as extensions of rate models such as the Linear-Nonlinear Poisson model where the activity of a homogeneous population is 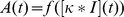 where 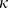 is a linear filter and 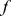 some nonlinear function. First, we focus on adaptive formulations of such rate models. For example Benda and Herz [15] have suggested that the firing rate of adapting neurons is a non-linear function of an input that is reduced by the past activity, such that the activity is 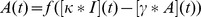 where 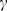 is a self interaction filter that summarizes the effect of adaptation. Second, we compare our approach with renewal theory [3], [42] which includes refractoriness, but not adaptation. How does our QR theory relate to these existing theories? And how would these competing theories perform on the same set of step stimuli?

To discuss the relation to existing theories, we recall that the instantaneous rate of our model 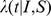 depends on both the input and the previous spike trains. In QR theory, we single out the most recent spike at 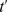 and averaged over the remaining spike trains 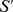: 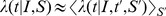. There are two alternative approaches. One can keep the most recent spike at 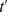 and disregard the effect of all the others: 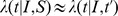. This gives rise to the time-dependent renewal theory, which will serve as a first reference for the performance comparison discussed below. On the other hand, one can average over *all* previous spikes, that is, no special treatment for the most recent one. In this case
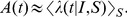
(9)The right-hand side of Eq. 9 can be treated with a moment expansion similar to the one in Eq. 7. To zero order, this gives a population rate 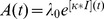, that is, an instantiation of the LNP model. To first order in an event-based moment expansion (EME1) we find:
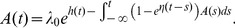
(10)Therefore, the moment expansion (Eq. 7) offers a way to link the phenomenological framework of Benda and Herz (2003) to parameters of the SRM. In particular, the nonlinearity is the exponential function, the input term is 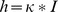 and the self-inhibition filter is 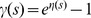. We note that Eq. 10 is a self-consistent equation for the population activity valid in the limit of small coupling between the spikes which can be solved using standard numerical methods (see Methods). A second-order equation (EME2) can similarly be constructed using an approximation to the correlation function (see Methods).

**Figure 5:**
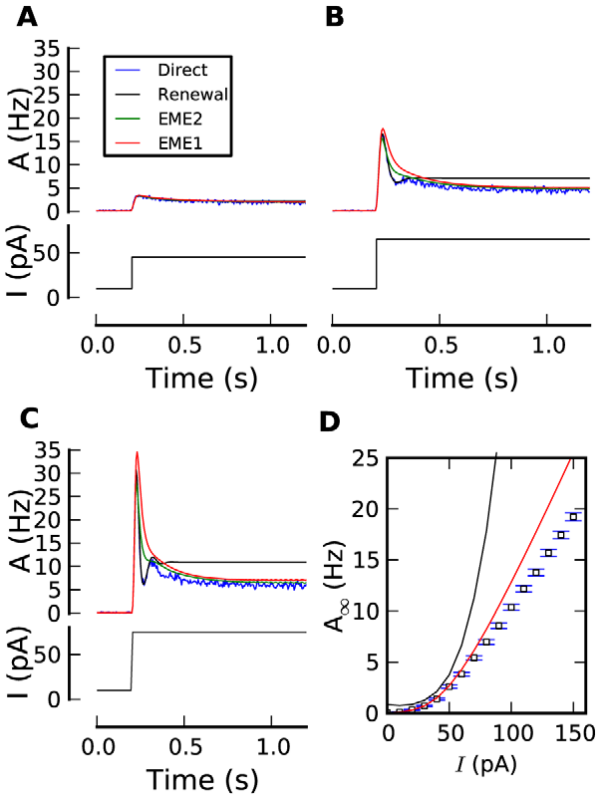
Approximative theories. (**A**–**C**) Population activity responses (top panels; PSTH from 25,000 repeated simulations in blue, renewal theory in black, first order moment expansion (EME1) in red, second order (EME2) in green) to the step current input (bottom panels; black). (**D**) Activity at the steady state vs. input current as calculated from the direct simulation of 25,000 model neurons (blue squares, error bars show one standard error of the mean), prediction from renewal theory (black), and 1st order moment-expansion (red, Eq. 51).

We compare the prediction of EME1, EME2 and renewal theory with the simulated responses to step inputs (Fig. 5). All the encoding frameworks work well for small input amplitudes (Fig. 5A). It is for larger input steps that the different theories can be distinguished qualitatively (Fig. 5C). Renewal theory predicts accurately the initial damped oscillation as can be expected by its explicit treatment of the relative refractory period. The adapting tail, however, is missing. The steady state is reached too soon and at a level which is systematically too high. EME1 is more accurate in its description of the adapting tail but fails to capture the damped oscillations. The strong refractory period induces a strong coupling between the spikes which means that truncating to only the first moment is insufficient. The solution based on EME2 improves the accuracy upon that of EME1 so as to make the initial peak shorter, but oscillates only weakly. We checked that the failure of the moment-expansion approach is due to the strong refractory period by systematically modifying the strength of the SAP (Fig. S2). Similarly, when the SAP is weak, the effect of 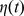 will often accumulate over several spikes and renewal theory does not capture the resulting adaptation (Fig. S2).

Fluctuating input makes the population respond in peaks of activity separated by periods of quiescence. This effectively reduces the coupling between the spikes and therefore improves the accuracy of EME1. The validity of EME1 for encoding time-dependent stimulus (Fig. S3) decreases with the STD of the fluctuating input with no clear dependence on the input mean.

Decoding with EME1 is done according to a simple relation:
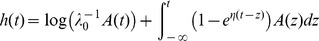
(11)where the logarithm of the momentary population activity is added to an accumulation of the past activity. The presence of the logarithm reflects the non-linearity for encoding (the link-function in Eq. 3) and leads to the fact that when the instantaneous population activity is zero, the stimulus is undefined but bounded from above: 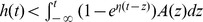. Fig. S4 shows the ability of Eq. 11 to recover the input from the population activity of 25,000 model neurons. We conclude that Eq. 11 is a valid decoder in the domain of applicability of EME1.

In summary, the EMEs yield theoretical expressions for the time-dependent as well as steady-state population activity. These expressions are valid in the limit of small coupling between the spikes which corresponds to either large interspike intervals or small SAP. Renewal theory on the other hand is valid when the single-neuron dynamics does not adapt and whenever the refractory effects dominate.

## Discussion

The input-output function of a neuron population is sometimes described as a linear filter of the input [41], as a linear filter of the input reduced as a function of past activity [58], [59], as a non-linear function of the filtered input [60], or by any of the more recent population encoding frameworks [47], [48], [61]–[65]. These theories differ in their underlying assumptions. To the best of our knowledge, a closed-form expression that does not assume weak refractoriness or weak adaptation has not been published before.

We have derived self-consistent formulas for the population activity of independent adapting neurons. There are two levels of approximation, EME1 (Eq. 10) is valid at low coupling between spikes which can be observed in real neurons whenever (i) the interspike intervals are large, (ii) the SAPs have small amplitudes or (iii) both the firing rate is low and the SAPs have small amplitudes. The second level of approximation merges renewal theory with the moment-expansion to give an accurate description on all time-scales. We called this approach the QR theory.

The QR equation captures almost perfectly the population code for time-dependent input even at the high firing rates observed in retinal ganglion cells [55]. But for the large interspike intervals and lower population activity levels of *in vivo* neurons of the cortex [66], [67], it is possible that the simpler encoding scheme of Eq. 10 is sufficient. Most likely, the appropriate level of approximation will depend on the neural system; cortical sparse coding may be well represented by EME, while neuron populations in the early stages of perception may require QR.

We have focused here on the Spike Response Model with escape noise which is an instantiation of a Generalized Linear Model. The escape noise model, defined as the instantaneous firing rate 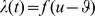 given the momentary distance between the (deterministic) membrane potential and threshold should be contrasted with the diffusive noise model where the membrane potential fluctuates because of noisy input. Nevertheless, the two noise models have been linked in the past [51], [54], [68]. For example, the interval-distribution of a leaky integrate-and-fire model with diffusive noise and arbitrary input can be well captured by escape noise with instantaneous firing rate 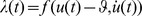 which depends both on the membrane potential and its temporal derivative 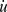 [51]. The dependence upon 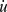 accounts for the rapid and replicable response that one observes when an integrate-and-fire model with diffusive noise is driven in the supra-threshold regime [68] and can, in principle, be included in the framework of the QR theory.

The decoding schemes presented in this paper (Eq. 11 and 45) reveal a fundamental aspect of population coding with adapting neurons. Namely, the ambiguity introduced by the adaptation can be resolved by considering a well-tuned accumulator of past activity. The neural code of adapting populations is ambiguous because the momentary level of activity could be the result of different stimulus histories. We have shown that resolving the ambiguity requires the knowledge of the activity in the past but to a good approximation does not require the knowledge of which neuron was active. At high population activity for neurons with large SAPs, however, the individual timing of the last spike in the spike trains is required to resolve the ambiguity (compare also Fairhall *et al.* [13]). Unlike bayesian spike-train decoding [55], [69], [70], we note that in our decoding frameworks the operation requires only knowledge of the population activity history and the single neuron characteristics. The properties of the QR or EME1 decoder can be used to find biophysical correlates of neural decoding such as previously proposed for short term plasticity [71], [72], non-linear dendrites [73] or lateral inhibition [74]. Note that, a constant percept in spite of spike frequency adaptation does not necessarily mean that neurons use a QR decoder. It depends on the synaptic structure. In an over-representing cortex, a constant percept can be achieved even when the neurons exhibit strong adaptation transients [75].

**Figure 6:**
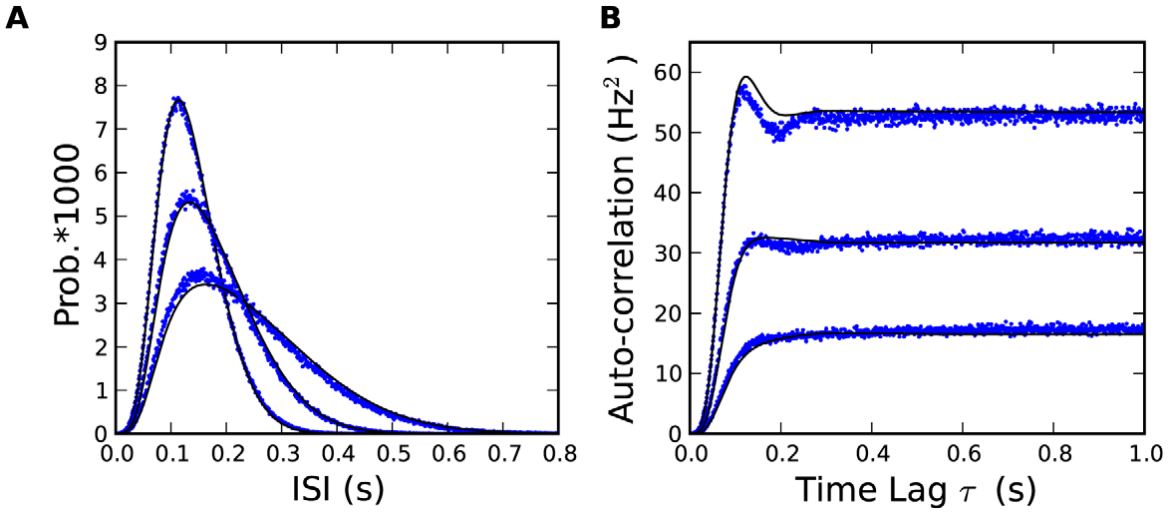
Steady-state interspike interval distribution and auto-correlation. (**A**)The interspike interval distribution calculated from the 25,000 repeated simulations of the SRM after the steady state has been reached (blue) is compared with the QR theory (Eq. 31; black) for I = 60, 70 and 80 pA. (**B**) On the same regimen, the autocorrelation function calculated from direct simulations at the steady-state (blue) is compared with the QR prediction (Eq. 33; black). See Methods for model parameters.

Using the results presented here, existing mean-field methods for populations of spiking neurons can readily be adapted to include spike-frequency adaptation. In Methods we show the QR theory for the interspike interval distribution and the steady-state autocorrelation function (Fig. 6) as well as linear filter characterizing the impulse response function (or frequency-dependent gain function) of the population. From the linear filter and the autocorrelation function, we can calculate the signal-to-noise ratio [3] and thus the transmitted information [1]. The autocorrelation function also gives an estimate of the coefficient of variation [76] and clarifies the role of the SAP in quenching the spike count variability [49], [77], [78]. The finite-size effects [27], [79]–[81] is another, more challenging, extension that should be possible.

The scope of the present investigation was restricted to unconnected neurons. In the mean-field approximation, it is straight-forward to extend the results to several populations of connected neurons [6]. For instance, similar to EME1, a network made of inter-connected neurons of 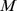 cell-types would correspond to the self-consistent system of equation:
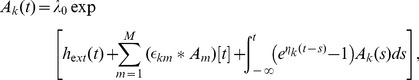
(12)where 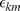 is the scaled post-synaptic potential kernel from cell-type 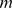 to cell-type 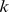 (following the formalism of Gerstner and Kislter [3]), 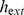 is an external driving force, each subpopulation is characterized by its population activity 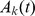 and its specific spike after potential 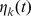. The analogous equation for QR theory is:
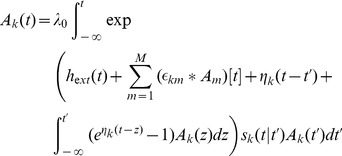
(13)where 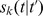 is:
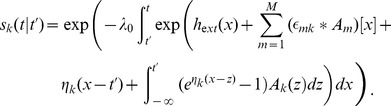
(14)Since the SAP is one of the most important parameter for distinguishing between cell classes [22], the approach presented in this paper opens the door to network models that take into account the neuronal cell-types beyond the sign of the synaptic connection. Even within the same class of cells, real neurons have slightly different parameters from one cell to the next [22] and it remains to be tested whether we can describe a moderately inhomogeneous population with our theory. Also, further work will be required to see if the decoding methods presented here can be applied to brain-machine interfacing [82]–[84].

## Methods

This section is organized in 3 subsections. Subsection A covers the mathematical steps to derive the main theoretical results (Eqs. 2, 5 and 7). It also presents a new approach to the time-dependent renewal equation, links with renewal theory and the derivation of the steady-state interspike interval distribution and auto-correlation. Subsection B covers the numerical methods and algorithmic details and subsection C the analysis methods.

### A Mathematical Methods

#### Derivation of Eq. 2

The probability density of a train of 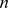 spikes 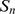 in an interval 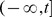 is given by [85]:
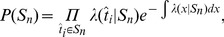
(15)where we omit writing the dependence on the input 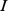 for notational convenience. Here 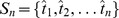 is the spike train where 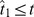 denotes the most recent spike, 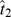 the previous one and so on. Instead of 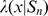 we can also write 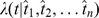. Note that because of causality, at a time 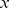 with 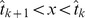, 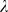 can only depend on *earlier* spikes so that 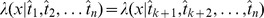. Special care has to be taken because of the discontinuity of 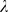 at the moment of the spike. We require 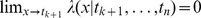 so that it is excluded that two spikes occur at the same moment in time. By definition, the population activity is the expected value of a spike train: 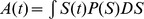. Following van Kampen [57] we can integrate over all possible spike times in an ordered or non-ordered fashion. In the ordered fashion, each spike time 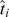 is restricted to times before the next spike time 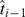. We obtain:
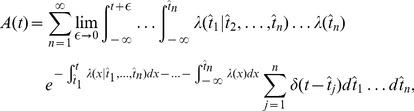
(16)where the term 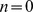 has been eliminated by the fact that 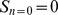. The notation 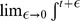 is intended to remind the reader that a spike happening exactly at time 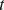 is included in the integral. In fact only *one* Dirac-delta function gives a non-vanishing term because only the integral over 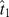 includes the time 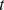. After integration over 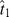 we have:
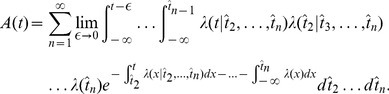
(17)Note that there are now 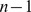 integrals and the first integral is over 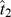 with an upper limit at 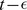. The 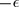 makes clear that the spike 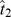 must be before the spike at 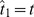. In the ordered notation 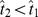. Re-labelling the infinite sum with 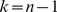, one readily sees that we recover the weighting factor 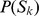 of a specific spike train with 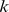 spikes (Eq. 15) in front of the momentary firing intensity 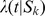:
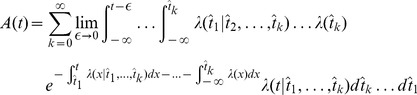
(18)Therefore we have shown Eq. 2, which we repeat here in the notation of the present paragraph:
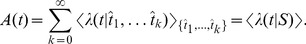
(19)Note that the term with zero spikes in the past (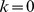) contributes a term 
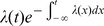
 to the sum.

#### Derivation of Eq. 5

In order to single out the effect of the previous spike, we replace 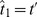 and group factors in the path integral of Eq. 18:
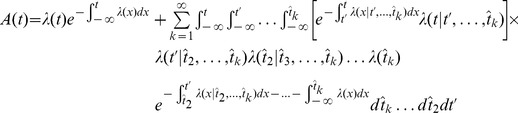
(20)The first term contains the probability that no spike was ever fired by the neuron until time 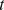. We can safely assume this term to be zero. The factors in square brackets depend on all previous spike times. However if we assume that the adaptation effects only depend on the most recent spike time 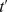 and on the typical spiking history before, but not on the specific spike times of earlier spikes, then the formula in square brackets can be moved in front of the integrals over 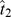, 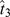, … We therefore set:
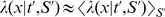
(21)where 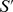 is the spike train containing all the spikes before 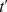. Thus, 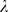 is now only a function of 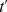 but not of the exact configuration of earlier spikes. We use the approximation of Eq. 21 only for the factors surrounded by square brackets in Eq. 20. The path integral Eq. 20 becomes:
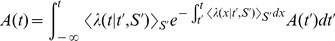
(22)where we have used Eq. 17 to recover 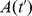.

#### Derivation of Eq. 7

We can recognize in 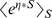 the moment generating functional for the random function 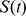. This functional can be written in terms of the correlation functions such as 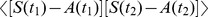 [57]. The correlation functions are labeled 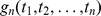 as in van Kampen [57] such that the first correlation function is the population activity: 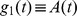, the second correlation function is 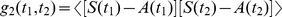 for 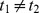, and so on. Then, the generating functional can be written [57]:
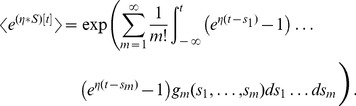
(23)
Eq. 23 is called a generating functional because the functional derivatives with respect to 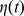 and evaluated at 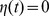 yields the correlation functions.

#### Derivation of the renewal equation

A derivation of the renewal equation [6], [41], [42] can be obtained by replacing the QR approximation (Eq. 21) by the renewal approximation:
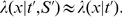
(24)Applying this approximation on the factors in the square bracket of Eq. 20 gives:
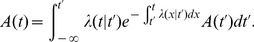
(25)Therefore Eqs. 20 and 24 yield a novel path integral proof of the renewal equation (Eq. 25).

#### The survival function and interval distribution

First consider the expected value in Eq. 2 partitioned so as to first average over the previous spike 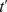 and then over the rest of the spiking history 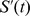:
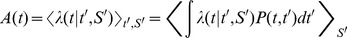
(26)where the last equality results from a marginalization of the last spike time. 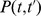 is the probability to spike at time 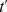 *and* to survive from 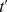 to 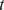 without spiking. Thus we can write 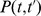 as the product of the population activity at 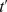 and the probability of not spiking between 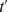 and 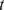 that we will label 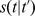:
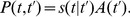
(27)The function 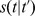 is the survival function in renewal theory. It depends implicitly on the spiking history. The rate of decay of the survival function depends in general on the precise timing of *all* previous spikes. The QR approximation means that we approximate this decay by averaging over all possible spike trains before 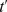, so that:
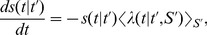
(28)which can be integrated to yield:
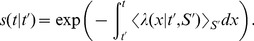
(29)The survival function in Eq. 27 and Eq. 26 leads to the QR equation (Eq. 5). Following renewal theory [3], the interspike interval distribution:
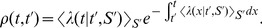
(30)The factor in Eq. 5 can therefore be interpreted as an approximate expression of the interspike interval distribution of adaptive neurons.

#### Auto-correlation functions and interspike interval distributions at the steady state

At the steady state with a constant input 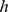, the interspike interval distribution predicted by QR theory is:
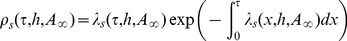
(31)where 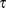 is the interspike interval, 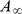 is the steady-state activity, and 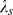 is the averaged conditional firing intensity 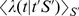. The latter can be written as:
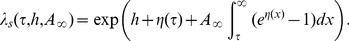
(32)From which we recover the auto-correlation function 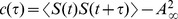 [3]:
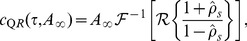
(33)where 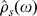 is the Fourier transform of 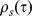. To solve for the steady-state population activity, we note that the inverse of 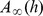 is also the mean interspike interval at the steady state:
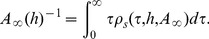
(34)


### B Numerical Methods

All simulations were performed on a desktop computer with 4 cores (Intel Core i7, 2.6 GHz, 24 GB RAM) using Matlab (The Mathworks, Natwick, MA). The Matlab codes to numerically solve the self-consistent equations are made available on the author's websites. The algorithmic aspects of the numerical methods are discussed now.

#### Direct simulation

All temporal units in this code are given in milliseconds. Direct simulation of Eq. 3 was done by first discretizing time (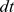 was varied between 0.5 and 0.005 ms) and then deciding at each time step whether a spike is emitted by comparing the probability to spike in a time-bin:
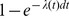
(35)to a random number of uniform distribution. Each time a spike is emitted, the firing probability is reduced according to the SRM equation for 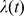 because another term 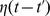 is added (Eq. 3). Typically 25,000 repeated simulations were required to compute PSTHs on such a fine temporal resolution. The PSTHs were built by averaging the 25,000 discretized spike trains and performing a 2-ms running average unless otherwise mentioned. The dynamics of 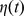 were calculated from the numerical solution of the differential equation corresponding to 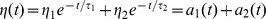 where 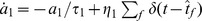 and similarly for 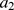.

For all simulations, the baseline current was 10 pA (except for time-dependent current where the mean was specified), the baseline excitability was 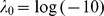 kHz, the membrane filter 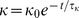 was a single exponential with an amplitude 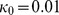 in units of inverse electric charge and a time constant of 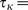
10 ms.

Time-dependent input consisted of an Ornstein-Uhlenbeck process which is computed at every time step as:
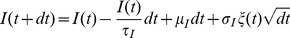
(36)where 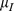 is the mean, 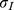 the standard deviation and 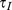 = 300 ms the correlation time constant. 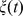 is a zero mean, unit variance Gaussian variable updated at every time step.

#### Numerical solution of renewal and quasi-renewal equations

We consider the QR equation, Eq. 5, with the averaged conditional intensity of Eq. 8. We choose a tolerance 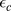 for truncating the function 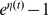 and find the cutoff 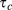 such that: 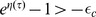 for all 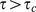. A typical value for the tolerance, 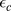, is 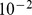. We split the main integral in Eq. 5 in two integrals, one from 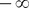 to 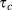, the other from 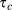 to 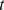 to get:
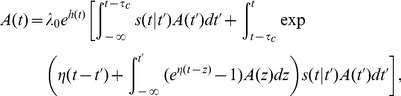
(37)where 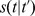 is called the survival function (see Methods A) and corresponds to 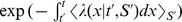. With the same reasoning the lower bound of the innermost integral can be changed from 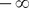 to 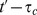 because 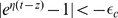 for all 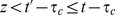. The first term in the square brackets of Eq. 37 are the neurons that have had their last spike a long time back in the past. For this term, we use the conservation equation, 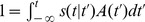 [3]. This enables us to write the first-order QR equation in terms of an integral from 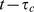 to 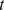 or 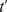 only:
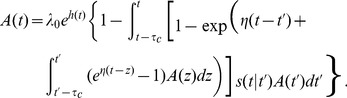
(38)We define two vectors. First 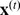 is made of the exponential in Eq. 38 on the linear grid for 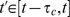, such that the 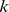
'th element can be written:
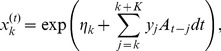
(39)where 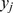 is the discretized function 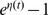. 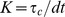 is the number of time steps 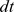 needed to cover the time span 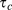 defined above. Note that 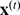 does not depend on 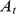 since 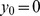 (because of an absolute refractoriness during the spike). The update of 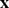 is the computationally expensive step of our implementation. Adaptive time-step procedures could be applied to improve the efficiency of the algorithm, but we did not do so. The special case where a rapid solution is possible is discussed further below.

The second vector 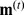 corresponds to 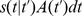 for 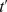 evaluated on the same linear grid as the one used for 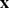. This vector traces the probability of having the last spike at 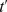. Assuming that there was no stimulation before time 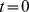 we can initialize this vector to zero. To update 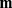 we note that the firing condition Eq. 35 gives:
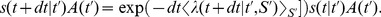
(40)To do so, we see from Eq. 40 that we can evaluate 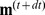 from 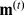 calculated at the previous time step. The first bin is updated to the previous population activity:
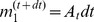
(41)and all the other bins are updated according to
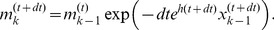
(42)


We can therefore calculate the population activity iteratively at each time bin using Eq. 38:
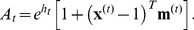
(43)where 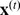 and 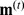 depend on the activity 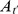 for 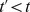. This algorithm implements a numerical approximation of the QR equation. On our desktop computer and with our particular implementation, solving 1 second of biological time took 36 seconds with a discretization of 0.1 ms for QR and 84–200 seconds for direct simulation of 25,000 neurons, depending on the firing rate. Using the same number of neurons but with a discretization of 0.5 ms it took 1.8 seconds to solve QR and 16–20 seconds to perform the direct simulation. If 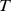 is the total number of time step, the present numerical methods are 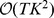. Evaluating the convolution in Eq. 39 with fast Fourier transform gives 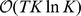. This same convolution can be evaluated with a differential equation with the choice of basis: 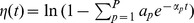, with parameters 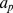 and 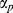 having the constraint of 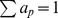. This fast parametrization solves in 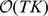.

#### Decoding QR

Isolating the input 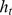 from Eq. 43 gives the decoding algorithm:
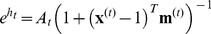
(44)where 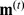 is also a function of 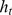. Decoding can be carried out by assuming 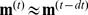 in Eq. 44, but this can lead to numerical instabilities when the time step is not very small. Instead we write 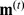 as a function of 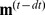 (Eqs. 41 and 42), expand Eq. 42 to first order in 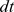 and solve the resulting quadratic equation for 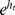:
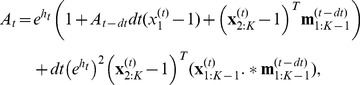
(45)where 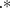 denotes the element by element (array) multiplication.

#### Numerical solution of EME1 and EME2

The structure of EME1 and EME2 allows us to use a nonlinear grid spacing in order to save memory resources. The bins should be small where 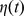 varies fast, and larger where 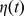 varies slowly. Since the SAP is approximatively exponential, we choose the size 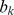 of bin 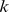 to be given by: 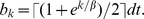 where 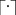 takes the nearest greater integer and 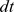 is the smallest time bin allowed and will be the discretization of the final solution for the population activity. The degree of nonlinearity, 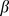, is chosen such that there are 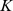 bins between 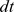 and 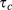. To a good approximation, 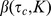 can be obtained by solving the equation: 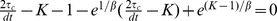.

To perform the numerical integration, we define the vector 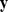 made of the function 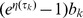 evaluated at the end of each bin 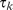 with bin size 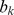, the vector 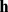 with elements 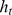 made of the convolution 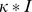 discretized on the uniform grid of length 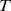 with bin size 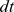, and on the same grid the vector 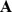 made of the discretized population activity. Finally, we define the vector 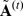 made of the population activity in the last 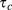 seconds since time 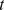 on the non-linear grid defined by 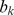. Using the rectangle method to evaluate the integral of the first-order self-consistent equation for population activity, we can write:
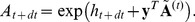
(46)Such that the population activity is obtained by solving iteratively through time Eq. 46, an operation requiring 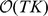.

To compute the second order equation, we first build the correlation vector 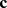 on the linear grid of the smallest bin size 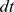:
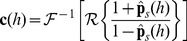
(47)where 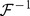 denotes the inverse Fourier transform and 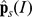 is the Fourier transform of 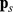, the steady-state interspike interval distribution for a renewal process. The steady-state inter-spike interval distribution vector is calculated from:
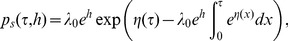
(48)where 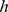 is a constant input and 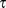 is an interspike interval. We assume that at each time 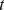 the correlation function is the steady-state correlation function associated with 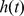. Then we construct the matrix 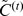 such that its element 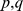 can be written:
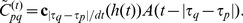
(49)Since the logarithmically spaced 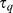 were multiples of 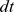 this matrix can be computed from 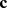. We first construct a look-up table for the correlation function for a range of the filtered input 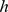. This way the matrix 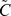 can be easily computed at each time step by updating with the new values of the population activity 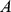. Finally, we evaluate the self-consistent equation of the population activity with the second order correction:
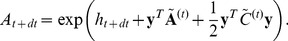
(50)


#### EME1 gain function

The first-order expansion (Eq. 10) can be used to write an analytical expression for the steady-state population activity. A constant input 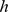 will bring the neuron population to a constant population activity that is obtained by solving for the constant 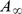 in Eq. 10.
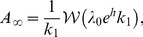
(51)where 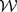 is the Lambert W-function and 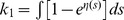. This gain function is valid on a restricted range of input (Fig. 5D).

### C Analysis Methods

When assessing the accuracy of the encoding or the decoding, we used the correlation coefficient. The correlation coefficient is the variance-normalized covariance between two random variables 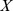 and 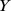:
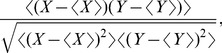
(52)where the expectation is taken over the discretized time.

## Supporting Information

Figure S1
**Statistics of decoding performance.** (**A**–**B**) Correlation coefficient between original filtered input recovered from the activity of a population of 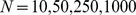 or 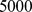 neurons shown as a function of 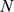. The activity was filtered with a given single exponential filter with a time constant of (**A**) 20 ms and (**B**) 2 ms. (**C**) Mean squared error associated with an instantaneous firing rate (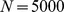, error bars correspond to one standard deviation). (**D**–**E**) Fraction of input times at which decoding could be performed corresponding to **A** and **B**, respectively. Decoding could not be carried out when the stimulus was outside the dynamic range which corresponds to 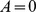. (**F**) Fraction of times where the activity was non-zero as a function of the population size. Colors show different standard deviation of the original input with values in pA, other parameters idem as Fig. 4.(TIF)

Figure S2
**Role of SAP for Renewal theory, EME1 and EME2 for step input.** Population activity responses (top panels; PSTH from 25,000 repeated simulations in blue, renewal theory in black, EME1 in red, EME2 in green) to the step current input (bottom panels; black). The neuron population follows spike-response model dynamics with effective SAP 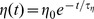 with 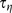 = 500 ms. (**A–C**) shows exemplar traces for different SAP amplitude and input steps: (**A**) 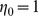 and current step 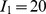 pA, (**B**) 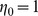 and current step 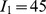 pA, (**C**) 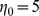 and current step 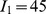 pA. The mean square error of each analytical approximation (**D** Renewal, **E** EME1, **F**, EME2) for various values of the SAP amplitude 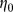 and current step size 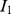. The error rate is the standard deviation between the PSTH and the theory as calculated on the first 2 seconds after the step, divided by 2 seconds. For other model parameters see Methods.(TIF)

Figure S3 **Encoding time-dependent stimuli in the population activity with Event-Based Moment Expansion (EME).** (**A**) Population activity responses (middle panel; PSTH from 25,000 repeated simulations in blue, EME1 in red to the time-dependent stimuli (bottom panel; black). The difference between direct simulation and theory is shown in the top panel.The stimulus is an Ornstein-Uhlenbeck process with correlation time constant of 300 ms with STD increasing every 2 seconds (20,40,60 pA) and a mean of 10 pA. (**B**) Correlation coefficients between direct simulation and EME1 for various STDs and mean (in pA) of the input current. Results of Fig. 3 are copied (dashed lines).
(TIF)

Figure S4
**Decoding the stimulus from the population activity with EME1.** (**A**–**D**) The original (bottom panels, black line) and decoded stimulus (bottom panels, red line; arbitrary units) recovered from the PSTH of 25,000 independent SRM neurons (top panels; blue line) using Eq. 11. The decoded waveform of negative input is occasionally undefined because the logarithm of zero activity is not defined (Eq. 11). (**E**) Correlation coefficient of original and decoded input as a function of input STD, shown for three distinct mean input (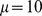 pA, 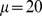 pA, and 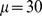 pA). Compare also with QR in Fig. 4.(TIF)

## References

[1] Rieke F, Warland D, de Ruyter van Steveninck R, Bialek W (1996) Spikes - Exploring the neural code. Cambridge: MIT Press.

[2] Dayan P, Abbott LF (2001) Theoretical Neuroscience. Cambridge: MIT Press.

[3] Gerstner W, Kistler W (2002) Spiking neuron models. New York: Cambridge University Press.

[4] Thorpe S, Fize D, Marlot C (1996) Speed of processing in the human visual system. Nature 381: 520–522. 8632824 10.1038/381520a0

[5] Abeles M (1991) Corticonics. Cambridge: Cambridge University Press.

[6] Gerstner W (2000) Population dynamics of spiking neurons: Fast transients, asynchronous states, and locking. Neural Comput 12: 43–89. 10636933 10.1162/089976600300015899

[7] Averbeck B, Latham P, Pouget A (2006) Neural correlations, population coding and computation. Nat Rev Neurosci 7: 358–366. 16760916 10.1038/nrn1888

[8] Smith RL (1979) Adaptation, saturation, and physiological masking in single auditory-nerve fibers. J Acoust Soc Am 65: 166–78. 422812 10.1121/1.382260

[9] Baccus S, Meister M (2002) Fast and slow contrast adaptation in retinal circuitry. Neuron 36: 909–919. 12467594 10.1016/s0896-6273(02)01050-4

[10] Sclar G, Lennie P, DePriest DD (1989) Contrast adaptation in striate cortex of macaque. Vision Res 29: 747–55. 2623819 10.1016/0042-6989(89)90087-4

[11] Ringo JL (1996) Stimulus specific adaptation in inferior temporal and medial temporal cortex of the monkey. Behav Brain Res 76: 191–7. 8734053 10.1016/0166-4328(95)00197-2

[12] Laughlin SB, Sejnowski TJ (2003) Communication in neuronal networks. Science 301: 1870–4. 14512617 10.1126/science.1089662 PMC2930149

[13] Fairhall AL, Lewen G, Bialek W, van Steveninck R (2001) Efficiency and ambiguity in an adaptive neural code. Nature 412: 787–792. 11518957 10.1038/35090500

[14] Seriès P, Stocker AA, Simoncelli EP (2009) Is the homunculus “aware” of sensory adaptation? Neural Comput 21: 3271–304 doi:10.1162/neco.2009.09-08-869. 19686064 10.1162/neco.2009.09-08-869 PMC3134250

[15] Benda J, Herz A (2003) A universal model for spike-frequency adaptation. Neural Comput 15: 2523–2564. 14577853 10.1162/089976603322385063

[16] Storm JF (1987) Action potential repolarization and a fast after-hyperpolarization in rat hippocampal pyramidal cells. J Physiol 385: 733–759. 2443676 10.1113/jphysiol.1987.sp016517 PMC1192370

[17] Schwindt P, Spain W, Foehring R, Stafstrom C, Chubb M, et al (1988) Multiple potassium conductances and their functions in neurons from cat sensorimotor cortex in vitro. J Neurophysiol 59: 424. 3351569 10.1152/jn.1988.59.2.424

[18] Schwindt P, Spain W, Foehring R, Chubb M, Crill W (1988) Slow conductances in neurons from cat sensorimotor cortex in vitro and their role in slow excitability changes. J Neurophysiol 59: 450. 3351570 10.1152/jn.1988.59.2.450

[19] Hill A (1936) Excitation and accomodation in nerve. Proc Biol Sci 119: 305–355.

[20] Fuortes M, Mantegazzini F (1962) Interpretation of the repetitive firing of nerve cells. J Gen Physiol 45: 1163–1179. 13895926 10.1085/jgp.45.6.1163 PMC2195242

[21] Azouz R, Gray CM (2000) Dynamic spike threshold reveals a mechanism for synaptic coincidence detection in cortical neurons in vivo. Proc Natl Acad Sci U S A 97: 8110–5. 10859358 10.1073/pnas.130200797 PMC16678

[22] Mensi S, Naud R, Avermann M, Petersen CCH, Gerstner W (2012) Parameter extraction and classification of three neuron types reveals two different adaptation mechanisms. J Neurophysiol 107: 1756–1775. 22157113 10.1152/jn.00408.2011

[23] La Camera G, Rauch A, Thurbon D, Lüscher H, Senn W, et al (2006) Multiple time scales of temporal response in pyramidal and fast spiking cortical neurons. J Neurophysiol 96: 3448–3464. 16807345 10.1152/jn.00453.2006

[24] Amit DJ, Tsodyks MV (1991) Quantitative study of attractor neural networks retrieving at low spike rates. i: Substrate — spikes, rates, and neuronal gain. Network 2: 259–273.

[25] Gerstner W, van Hemmen J (1992) Universality in neural networks: the importance of the ‘mean firing rate’. Biol Cybern 67: 195–205. 1498186 10.1007/BF00204392

[26] Amit DJ, Brunel N (1997) A model of spontaneous activity and local delay activity during delay periods in the cerebral cortex. Cereb Cortex 7: 237–252. 9143444 10.1093/cercor/7.3.237

[27] Brunel N (2000) Dynamics of sparsely connected networks of excitatory and inhibitory spiking neuron. J Comput Neurosci 8: 183–208. 10809012 10.1023/a:1008925309027

[28] Renart A, de la Rocha J, Bartho P, Hollender L, Parga N, et al (2010) The asynchronous state in cortical circuits. Science 327: 587–90. 20110507 10.1126/science.1179850 PMC2861483

[29] Fourcaud-Trocme N, Hansel D, Vreeswijk CV, Brunel N (2003) How spike generation mechanisms determine the neuronal response to uctuating inputs. J Neurosci 23: 11628–11640. 14684865 10.1523/JNEUROSCI.23-37-11628.2003 PMC6740955

[30] Diesmann M, Gewaltig MO, Aertsen A (1999) Stable propagation of synchronous spiking in cortical neural networks. Nature 402: 529–533. 10591212 10.1038/990101

[31] Pillow J, Paninski L, Uzzell V, Simoncelli E, Chichilnisky E (2005) Prediction and decoding of retinal ganglion cell responses with a probabilistic spiking model. J Neurosci 25: 11003–11013. 16306413 10.1523/JNEUROSCI.3305-05.2005 PMC6725882

[32] Jolivet R, Rauch A, Luscher H, Gerstner W (2006) Predicting spike timing of neocortical pyramidal neurons by simple threshold models. J Comput Neurosci 21: 35–49. 16633938 10.1007/s10827-006-7074-5

[33] Jolivet R, Kobayashi R, Rauch A, Naud R, Shinomoto S, et al (2008) A benchmark test for a quantitative assessment of simple neuron models. J Neurosci Methods 169: 417–424. 18160135 10.1016/j.jneumeth.2007.11.006

[34] Hubel D, Wiesel T (1962) Receptive fields, binocular interaction and functional architecture in the cat's visual cortex. J Physiol 160: 106–154. 14449617 10.1113/jphysiol.1962.sp006837 PMC1359523

[35] Marmarelis PZ, Naka K (1972) White-noise analysis of a neuron chain: an application of the wiener theory. Science 175: 1276–8. 5061252 10.1126/science.175.4027.1276

[36] Enroth-Cugell C, Robson JG (1966) The contrast sensitivity of retinal ganglion cells of the cat. J Physiol 187: 517–52. 16783910 10.1113/jphysiol.1966.sp008107 PMC1395960

[37] Gerstner W (2001) Coding properties of spiking neurons: reverse- and cross-correlations. Neural Netw 14: 599–610. 11665756 10.1016/s0893-6080(01)00053-3

[38] Aviel Y, Gerstner W (2006) From spiking neurons to rate models: a cascade model as an approximation to spiking neuron models with refractoriness. Phys Rev E 73: 51908. 10.1103/PhysRevE.73.051908 16802968

[39] Schwartz O, Sejnowski TJ, Dayan P (2006) Soft mixer assignment in a hierarchical generative model of natural scene statistics. Neural Comput 18: 2680–2718. 16999575 10.1162/neco.2006.18.11.2680 PMC2915771

[40] Ostojic S, Brunel N (2011) From spiking neuron models to linear-nonlinear models. PLoS Comput Biol 7: e1001056 doi:10.1371/journal.pcbi.1001056. 21283777 10.1371/journal.pcbi.1001056 PMC3024256

[41] Wilson HR, Cowan JD (1972) Excitatory and inhibitory interactions in localized populations of model neurons. Biophys J 12: 1–24. 4332108 10.1016/S0006-3495(72)86068-5 PMC1484078

[42] Gerstner W (1995) Time structure of the activity in neural network models. Phys Rev E 51: 738–758. 10.1103/physreve.51.738 9962697

[43] Rauch A, Camera GL, Luscher H, Senn W, Fusi S (2003) Neocortical pyramidal cells respond as integrate-and-fire neurons to in vivo-like input currents. J Neurophysiol 90: 1598–1612. 12750422 10.1152/jn.00293.2003

[44] La Camera G, Rauch A, Lüscher H, Senn W (2004) Minimal models of adapted neuronal response to in vivo-like input currents. Neural Comput 16: 2101–2124. 15333209 10.1162/0899766041732468

[45] Treves A (1993) Mean-field analysis of neuronal spike dynamics. Network 4: 259–284.

[46] Muller E, Buesing L, Schemmel J, Meier K (2007) Spike-frequency adapting neural ensembles: beyond mean adaptation and renewal theories. Neural Comput 19: 2958–3010. 17883347 10.1162/neco.2007.19.11.2958

[47] Richardson MJE (2009) Dynamics of populations and networks of neurons with voltage-activated and calcium-activated currents. Phys Rev E 80: 021928. 10.1103/PhysRevE.80.021928 19792172

[48] Toyoizumi T, Rad K, Paninski L (2009) Mean-field approximations for coupled populations of generalized linear model spiking neurons with markov refractoriness. Neural Comput 21: 1203–1243. 19718814 10.1162/neco.2008.04-08-757

[49] Farkhooi F, Muller E, Nawrot MP (2011) Adaptation reduces variability of the neuronal population code. Phys Rev E 83: 050905. 10.1103/PhysRevE.83.050905 21728481

[50] Gerstner W, van Hemmen J, Cowan J (1996) What matters in neuronal locking? Neural Comput 8: 1653–1676. 8888612 10.1162/neco.1996.8.8.1653

[51] Plesser H, Gerstner W (2000) Noise in integrate-and-fire neurons: From stochastic input to escape rates. Neural Comput 12: 367–384. 10636947 10.1162/089976600300015835

[52] Gerstner W (2008) Spike-response model. Scholarpedia 3: 1343.

[53] Paninski L (2004) Maximum likelihood estimation of cascade point-process neural encoding models. Network 15: 243–262. 15600233

[54] Mensi S, Naud R, Gerstner W (2011) From stochastic nonlinear integrate-and-fire to generalized linear models. In: Shawe-Taylor J, Zemel RS, Bartlett P, Pereira F, Weinberger KQ, editors. Advances in Neural Information Processing Systems 24. Cambridge: MIT Press.

[55] Pillow J, Shlens J, Paninski L, Sher A, Litke A, et al (2008) Spatio-temporal correlations and visual signalling in a complete neuronal population. Nature 454: 995–999. 18650810 10.1038/nature07140 PMC2684455

[56] Cox DR (1962) Renewal theory. London: Methuen.

[57] van Kampen NG (1992) Stochastic processes in physics and chemistry. 2nd edition. Amsterdam: North-Holland.

[58] Hawkes AG (1971) Spectra of some self-exciting and mutually exciting processes. Biometrika 58: 83–90.

[59] Pernice V, Staude B, Cardanobile S, Rotter S (2011) How structure determines correlations in neuronal networks. PLoS Comput Biol 7: e1002059 doi:10.1371/journal.pcbi.1002059. 21625580 10.1371/journal.pcbi.1002059 PMC3098224

[60] Schwartz O, Pillow JW, Rust NC, Simoncelli EP (2006) Spike-triggered neural characterization. J Vis 6: 484–507 doi:10.1167/6.4.13. 16889482 10.1167/6.4.13

[61] Knight BW (2000) Dynamics of encoding in neuron populations: some general mathematical features. Neural Comput 12: 473–518. 10769319 10.1162/089976600300015673

[62] Shriki O, Hansel D, Sompolinsky H (2003) Rate models for conductance-based cortical neuronal networks. Neural Comput 15: 1809–1841. 14511514 10.1162/08997660360675053

[63] Richardson MJE, Brunel N, Hakim V (2003) From subthreshold to firing-rate resonance. J Neurophysiol 89: 2538–2554. 12611957 10.1152/jn.00955.2002

[64] Richardson MJE (2007) Firing-rate response of linear and nonlinear integrate-and-fire neurons to modulated current-based and conductance-based synaptic drive. Phys Rev E 76: 021919. 10.1103/PhysRevE.76.021919 17930077

[65] Muller E, Buesing L, Schemmel J, Meier K (2007) Spike-frequency adapting neural ensembles: beyond mean adaptation and renewal theories. Neural Comput 19: 2958–3010. 17883347 10.1162/neco.2007.19.11.2958

[66] de Kock CPJ, Sakmann B (2009) Spiking in primary somatosensory cortex during natural whisking in awake head-restrained rats is cell-type specific. Proc Natl Acad Sci U S A 106: 16446–16450. 19805318 10.1073/pnas.0904143106 PMC2752569

[67] Crochet S, Poulet JFA, Kremer Y, Petersen CCH (2011) Synaptic mechanisms underlying sparse coding of active touch. Neuron 69: 1160–75. 21435560 10.1016/j.neuron.2011.02.022

[68] Herrmann A, Gerstner W (2001) Noise and the psth response to current transients: I. General theory and application to the integrate-and-fire neuron. J Computat Neurosci 11: 135–151. 10.1023/a:1012841516004 11717530

[69] Paninski L, Pillow J, Lewi J (2007) Statistical models for neural encoding, decoding, and optimal stimulus design. Prog Brain Res 165: 493–507. 17925266 10.1016/S0079-6123(06)65031-0

[70] Koyama S, Eden UT, Brown EN, Kass RE (2010) Bayesian decoding of neural spike trains. Ann Inst Stat Math 62: 37–59 doi:10.1007/s10463-009-0249-x.

[71] Sen K, Jorge-Rivera JC, Marder E, Abbott LF (1996) Decoding synapses. J Neurosci 16: 6307–6318. 8815910 10.1523/JNEUROSCI.16-19-06307.1996 PMC6579172

[72] Pfister JP, Dayan P, Lengyel M (2010) Synapses with short-term plasticity are optimal estimators of presynaptic membrane potentials. Nat Neurosci 13: 1271–5. 20852625 10.1038/nn.2640 PMC3558743

[73] Polsky A, Mel B, Schiller J (2009) Encoding and decoding bursts by nmda spikes in basal dendrites of layer 5 pyramidal neurons. J Neurosci 29: 11891–903. 19776275 10.1523/JNEUROSCI.5250-08.2009 PMC3850222

[74] Carandini M, Heeger D (1994) Summation and division by neurons in primate visual cortex. Science 264: 1333–1336. 8191289 10.1126/science.8191289

[75] Druckmann S, Chklovskii D (2010) Over-complete representations on recurrent neural networks can support persistent percepts. In: Lafferty J, Williams CKY, Shawe-Taylor J, Zemel RS, Culotta A, editors. Advances in Neural Information Processing Systems 24. Cambridge: MIT Press.

[76] Softky W, Koch C (1993) The highly irregular firing pattern of cortical cells is inconsistent with temporal integration of random epsps. J Neurosci 13: 334–350. 8423479 10.1523/JNEUROSCI.13-01-00334.1993 PMC6576320

[77] Liu YH, Wang XJ (2001) Spike-frequency adaptation of a generalized leaky integrate-and-fire model neuron. J Comput Neurosci 10: 25–45 doi:10.1023/A:1008916026143. 11316338 10.1023/a:1008916026143

[78] Wang XJ, Liu Y, Sanchez-Vives MV, McCormick DA (2003) Adaptation and temporal decorrelation by single neurons in the primary visual cortex. J Neurophysiol 89: 3279–93. 12649312 10.1152/jn.00242.2003

[79] Spiridon M, Gerstner W (1999) Noise spectrum and signal transmission trough a population of spiking neurons. Network 10: 257–272. 10496476

[80] Lindner B, Chacron M, Longtin A (2005) Integrate-and-fire neurons with threshold noise: A tractable model of how interspike interval correlations affect neuronal signal transmission. Phys Rev E 72: 021911. 10.1103/PhysRevE.72.021911 PMC5283900 16196608

[81] Buice MA, Chow CC (2007) Correlations, uctuations, and stability of a finite-size network of coupled oscillators. Phys RevE 76: 031118. 10.1103/PhysRevE.76.031118 17930210

[82] Chapin JK, Moxon KA, Markowitz RS, Nicolelis N (1999) Real-time control of a robot arm using simultaneously recorded neurons in the motor cortex. Nat Neurosci 2: 664–670 doi:10.1038/10223. 10404201 10.1038/10223

[83] Hatsopoulos NG, Donoghue JP (2009) The science of neural interface systems. Annu Rev Neurosci 32: 249–66. 19400719 10.1146/annurev.neuro.051508.135241 PMC2921719

[84] Koyama S, Chase S, Whitford A, Velliste M, Schwartz A, et al (2010) Comparison of brain–computer interface decoding algorithms in open-loop and closed-loop control. J Comput Neurosci 29: 73–87. 19904595 10.1007/s10827-009-0196-9

[85] Pfister J, Toyoizumi T, Barber D, Gerstner W (2006) Optimal spike-timing-dependent plasticity for precise action potential firing in supervised learning. Neural Comput 18: 1318–1348. 16764506 10.1162/neco.2006.18.6.1318

