# Supplementary figures and images for: Coding and Decoding with Adapting Neurons: A Population Approach to the Peri-Stimulus Time Histogram
Source: PLoS Comput Biol. 2012 Oct 4;8(10):e1002711. doi: 10.1371/journal.pcbi.1002711 (PMC3464223; doi:10.1371/journal.pcbi.1002711)

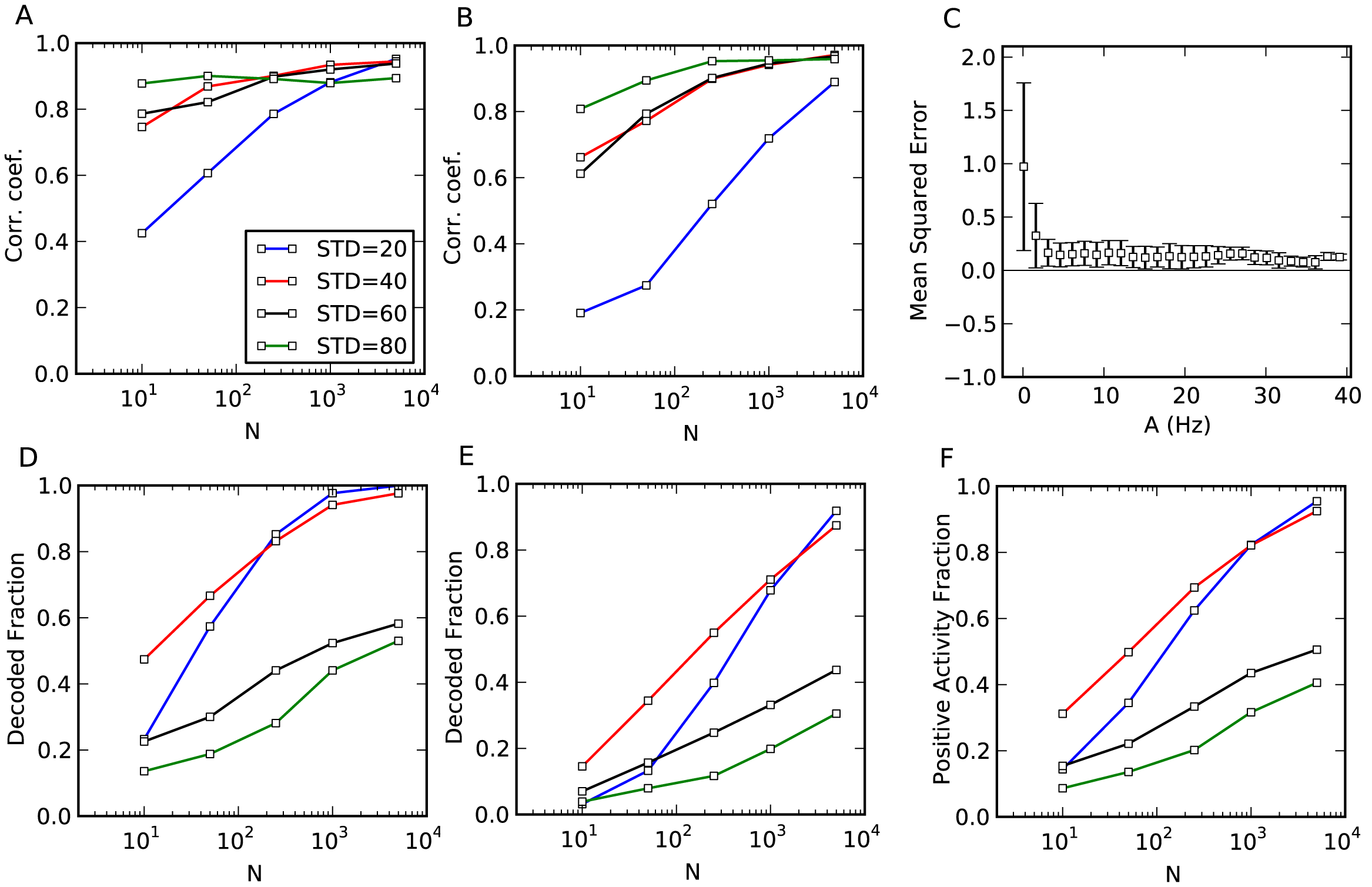

Supplement: Figure S1 — Statistics of decoding performance. (A–B) Correlation coefficient between original filtered input recovered from the activity of a population of or neurons shown as a function of . The activity was filtered with a given single exponential filter with a time constant of (A) 20 ms and (B) 2 ms. (C) Mean squared error associated with an instantaneous firing rate (, error bars correspond to one standard deviation). (D–E) Fraction of input times at which decoding could be performed corresponding to A and B, respectively. Decoding could not be carried out when the stimulus was outside the dynamic range which corresponds to . (F) Fraction of times where the activity was non-zero as a function of the population size. Colors show different standard deviation of the original input with values in pA, other parameters idem as Fig. 4. (TIF) [file pcbi.1002711.s001.tif]

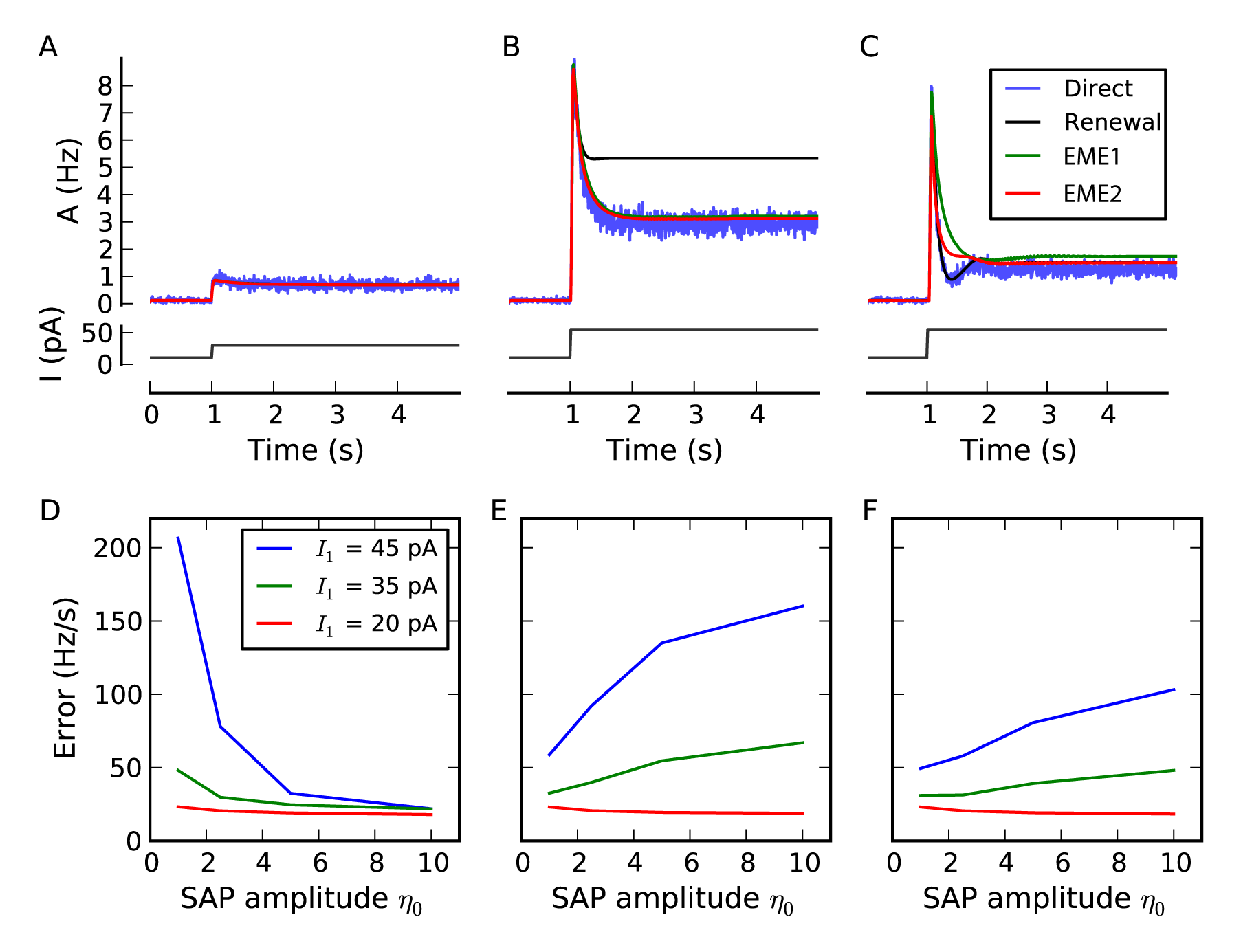

Supplement: Figure S2 — Role of SAP for Renewal theory, EME1 and EME2 for step input. Population activity responses (top panels; PSTH from 25,000 repeated simulations in blue, renewal theory in black, EME1 in red, EME2 in green) to the step current input (bottom panels; black). The neuron population follows spike-response model dynamics with effective SAP with = 500 ms. (A–C) shows exemplar traces for different SAP amplitude and input steps: (A) and current step pA, (B) and current step pA, (C) and current step pA. The mean square error of each analytical approximation (D Renewal, E EME1, F, EME2) for various values of the SAP amplitude and current step size . The error rate is the standard deviation between the PSTH and the theory as calculated on the first 2 seconds after the step, divided by 2 seconds. For other model parameters see Methods. (TIF) [file pcbi.1002711.s002.tif]

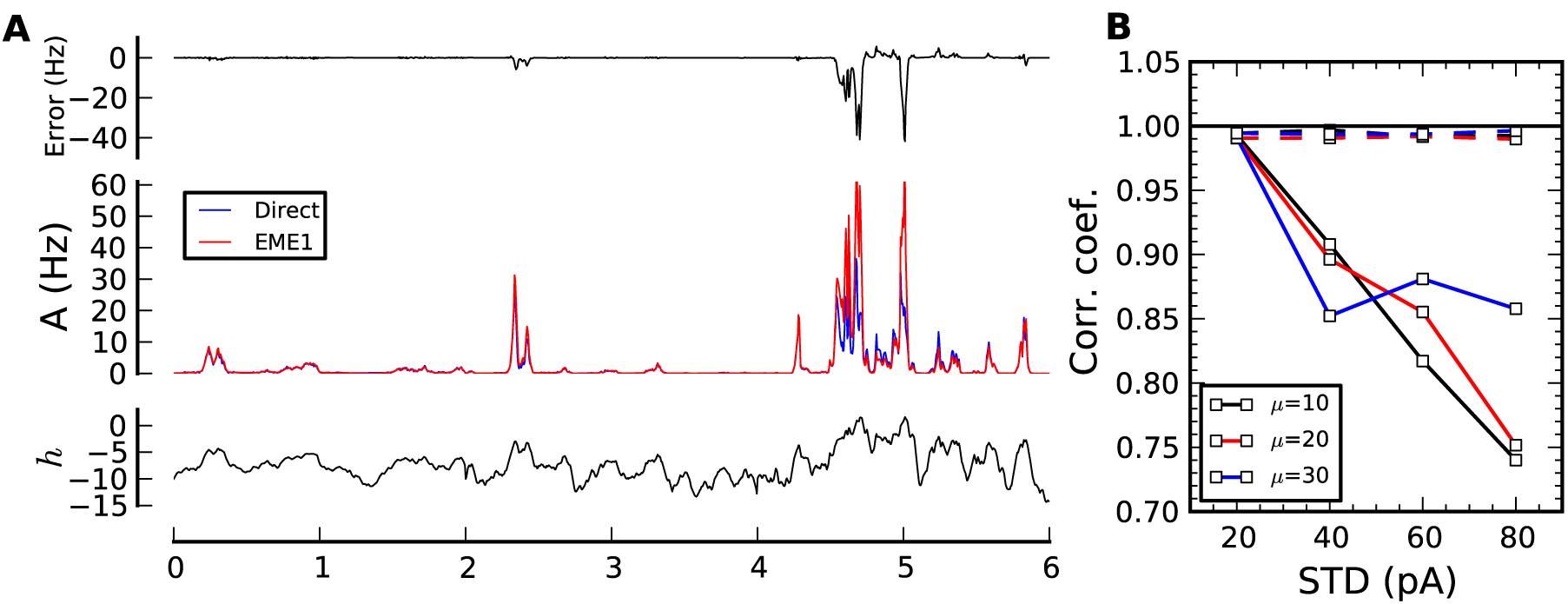

Supplement: Figure S3 — Encoding time-dependent stimuli in the population activity with Event-Based Moment Expansion (EME). (A) Population activity responses (middle panel; PSTH from 25,000 repeated simulations in blue, EME1 in red to the time-dependent stimuli (bottom panel; black). The difference between direct simulation and theory is shown in the top panel.The stimulus is an Ornstein-Uhlenbeck process with correlation time constant of 300 ms with STD increasing every 2 seconds (20,40,60 pA) and a mean of 10 pA. (B) Correlation coefficients between direct simulation and EME1 for various STDs and mean (in pA) of the input current. Results of Fig. 3 are copied (dashed lines). (TIF) [file pcbi.1002711.s003.tif]

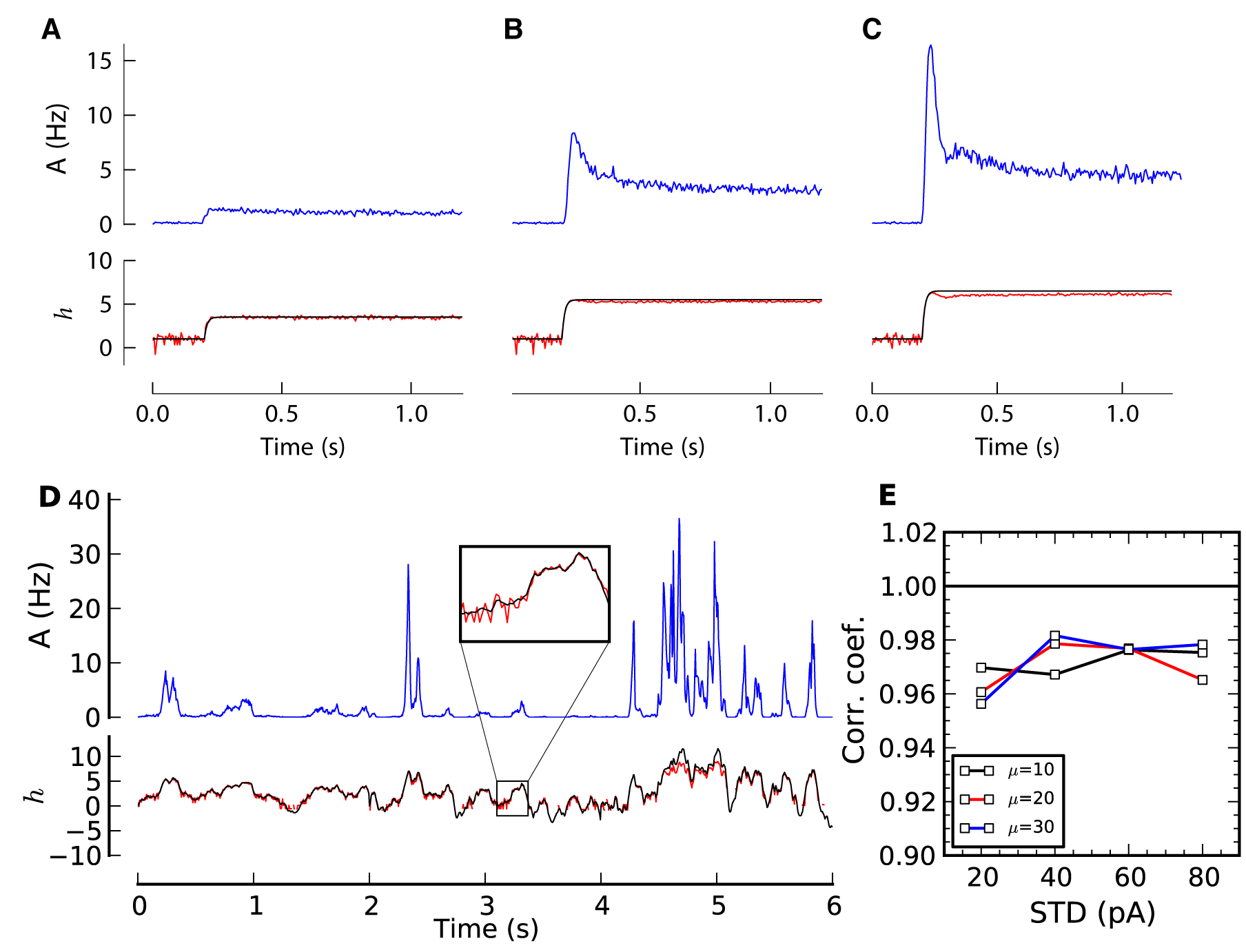

Supplement: Figure S4 — Decoding the stimulus from the population activity with EME1. (A–D) The original (bottom panels, black line) and decoded stimulus (bottom panels, red line; arbitrary units) recovered from the PSTH of 25,000 independent SRM neurons (top panels; blue line) using Eq. 11. The decoded waveform of negative input is occasionally undefined because the logarithm of zero activity is not defined (Eq. 11). (E) Correlation coefficient of original and decoded input as a function of input STD, shown for three distinct mean input ( pA, pA, and pA). Compare also with QR in Fig. 4. (TIF) [file pcbi.1002711.s004.tif]
